# Association of Pulmonary Tuberculosis and HIV in the Mexican Institute of Social Security, 2006-2014

**DOI:** 10.1371/journal.pone.0168559

**Published:** 2016-12-29

**Authors:** David Alejandro Cabrera-Gaytán, María del Rosario Niebla-Fuentes, Rosario Padilla-Velázquez, Gabriel Valle-Alvarado, Lumumba Arriaga-Nieto, Teresita Rojas-Mendoza, Ulises Rosado-Quiab, Concepción Grajales-Muñiz, Alfonso Vallejos-Parás

**Affiliations:** Coordinación de Vigilancia Epidemiológica, Instituto Mexicano del Seguro Social, Del Valle, Benito Juárez, México City, CP, México; Agencia de Salut Publica de Barcelona, SPAIN

## Abstract

**Background:**

Tuberculosis and HIV remain a public health problem in developed countries. The objective of this study was to analyze the incidence trends of pulmonary TB and HIV comorbidity and treatment outcomes according to HIV during the period 2006 to 2014 in the Mexican Institute of Social Security.

**Methods:**

Analyzed data from this registry including pulmonary tuberculosis patients aged 15 years and older who had been diagnosed during the years 2006 to 2014 in the Mexican Institute of Social Security. The outcomes that we use were incidents rate, failure to treatment and death. Regression models were used to quantify associations between pulmonary tuberculosis and HIV mortality.

**Results:**

During the study period, 31,352 patients were registered with pulmonary tuberculosis. The incidence rate observed during 2014 was 11.6 case of PTB per 100,000. The incidence rate for PTB and HIV was 0.345 per 100,000. The PTB incidence rate decreased by 0.07%, differences found in the PTB incidence rate by sex since in women decreased by 5.52% and in man increase by 3.62%. The pulmonary TB with HIV incidence rate decreased by 16.3% during the study period (In women increase 4.81% and in man decrease 21.6%). Analysis of PTB associated with HIV by age groups revealed that the highest incidence rates were observed for the 30 to 44 years old group. Meanwhile, the highest incidence rates of PTB without HIV occurred among the 60 and more years old individuals. We did not find statistically significant differences between treatment failure and PTB patients with HIV and without HIV. The treatment failure was associated with sex and the region of the patient. We found a strong association between HIV and the probability of dying during treatment. Our data suggested that patients suffering from both conditions (PTB and HIV) have no difference in the probability of failure of treatment contrary to other reports. Hypotheses to this is adherence to tuberculosis treatment with people living with HIV/AIDS, detection of PTB in patients suffering from HIV/AIDS or PTB patients on antiretroviral therapy were more likely to have successful treatment outcomes than those not on antiretroviral treatment. We have found that PTB and HIV increases the probability of dying during treatment compared to the cases of PTB without HIV, consistent with published other study HIV increases the mortality rates associated with PTB.

**Conclusions:**

No association between pulmonary tuberculosis with HIV and treatment failure was observed, but pulmonary tuberculosis and HIV increases the probability of dying during treatment compared to the pulmonary tuberculosis cases without HIV.

## Introduction

Tuberculosis (TB) remains one of the world’s deadliest communicable diseases. In 2013, an estimated 9.0 million people developed TB and 1.5 million died from the disease, 360 000 of whom were Human Immunodeficiency Virus (HIV) positive. TB is slowly declining each year and it is estimated that 37 million lives were saved between 2000 and 2013 through effective diagnosis and treatment. However, given that most deaths from TB are preventable, the death toll from the disease is still unacceptably high. People living with HIV who are also infected with TB are much more likely to develop TB disease than those who are HIV-negative. Starting in the 1980s, the HIV epidemic led to a major upsurge in TB cases and TB mortality in many countries[1].

For 2012, TB incidence rate in Mexico was of 23 per 100,000 inhabitants indicating that the disease continues to represent a public health problem [2].

In Mexico, the percentage of people infected with TB and Acquired Immune Deficiency Syndrome (AIDS) is estimated at 5.6% of all TB registers [3],and the incidence rate is 1 per 100,000 population [4].

Given the magnitude and clinical consequences of the association between TB and HIV in Mexico, the objective of this study was to analyze the incidence trends of pulmonary TB (PTB) and HIV comorbidity and treatment outcomes according to HIV during the period 2006 to 2014 at Mexican Institute of Social Security (IMSS).

## Methods

We conducted a retrospective analysis, we analyzed data from this registry including PTB patients aged 15 years and older who had been diagnosed during the years 2006 to 2014 in the IMSS.

In Mexico, all TB patients are mandatorily reported according to official guidelines and registered in the National Tuberculosis Registry in Mexico [5].

According to the official norms, TB patients were verified by acid fast bacilli (AFB) sputum smear, mycobacterial culture, histopathology, by clinical suspicion or radiological or association epidemiologic [5]. In our study, all patients were included, regardless of the diagnostic method, with at least one of them. Patients were treated under directly observed treatment, short-course (DOTS) strategy using the World Health Organization (WHO) standard regimen in which therapy was initiated with 4 drugs (2HRZE/4HR) for newly diagnosed patients. We used only new cases of PTB. The patient was considered to have HIV if she/he self-referred to have been previously diagnosed by a physician. This definition may underestimate patients who are unaware of their diagnosis; however, it is used for epidemiological purposes by the Mexican health surveys [6].

We used the same definitions use by Delgado-Sánchez, et al (Association of Pulmonary Tuberculosis and Diabetes in Mexico: Analysis of the National Tuberculosis Registry 2000–2012) [2]. The results of anti-tuberculosis treatment were defined according to official guidelines [5].

Briefly, failure was defined when AFB microscopies or cultures were positive at five months or later during treatment. Cure was defined when treatment was completed with the disappearance of signs and symptoms with two or more acid-fast bacilli smears or cultures with negative results at the end of therapy. Treatment completion was defined when a patient completed her/his treatment regimen with disappearance of signs and symptoms and smear or culture were not performed. Death was defined when a patient died of any cause during therapy. Treatment success was defined by the sum of patients who were cured and those who had completed treatment as defined above. Mexican guidelines have defined default when a patient interrupts treatment for 30 days or more rather than 60 days defined by WHO in order to be able to timely prevent that patients drop out from treatment.

We considered three regions, Mexico City and central Mexico, northern Mexico, and southern Mexico, according to regionalization used in the National Survey of Health and Nutrition 2012 [7].This regionalization has been used by previous epidemiological studies to compare different areas in the country and is based on common geographic and socioeconomic characteristics: (Northern region: Baja California, Baja California Sur, Coahuila, Chihuahua, Durango, Nuevo León, Sinaloa, Sonora, and Tamaulipas; Central Region: Aguascalientes, Colima, Guanajuato, Jalisco, México, Michoacán, Morelos, Nayarit, Querétaro, San Luis Potosí, Zacatecas, and Mexico City; and Southern Region: Campeche, Chiapas, Guerrero, Hidalgo, Oaxaca, Puebla, Quintana Roo, Tabasco, Tlaxcala, Veracruz, and Yucatán) [2].

Incidence rates were calculated using data from the TB registry as the numerator and the data from the insured population over 15 years old according to official data Mexican Social Security Institute as denominator [8].

We calculated percent change of annual trends overall and according to age group among patients with HIV, without HIV and overall.

Clinical characteristics of patients with and without information on previous HIV diagnosis were compared using Pearson's Chi. Associations between sociodemographic and clinical characteristics with PTB and HIV were tested by multivariate logistic regression. Associations between PTB and HIV and treatment failure, as well as associations between PTB and HIV and death, were investigated by multivariate logistic regression. All multivariate analyses accounted for sex, age and regional distribution. We estimated the odds ratios (OR) and 95 per cent confidence intervals (CI), and identified the covariates that were independently associated with each outcome.

## Results

During the study period, 31,352 patients were registered with PTB, (58.7% male and 41.3% female), 1,325 (4.2%) patients had HIV a previous diagnosis.

**Fig 1:**
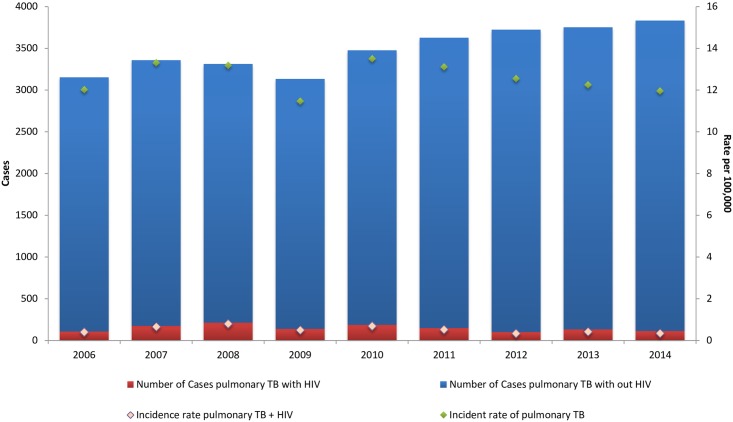
Trends of incidence rate of pulmonary TB and HIV and number of cases. IMSS, 2006–2014.

The incidence rate observed during 2014 was 11.6 case of PTB per 100,000; and incidence rate for PTB and HIV was 0.345 per 100,000. (Fig 1).

**Fig 2:**
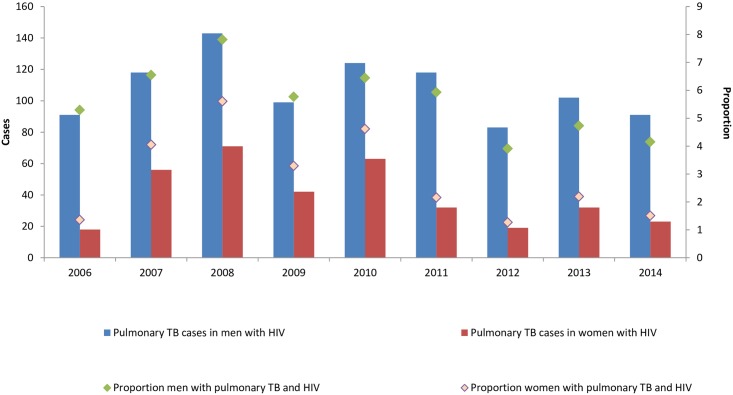
Trends of pulmonary TB and HIV by sex, cases and proportion, Mexican Institute of Social Security, 2006–2014.

During the study period, PTB incidence rate decreased by 0.07% but in women decreased by 5.52% and in man increase by 3.62%. The PTB with HIV rates decreased by 16.3% (in women increase 4.81% and in man decrease 21.6%). (Fig 2).

Statistical difference between the proportion of cases among men and women of PTB with HIV was found (p <0.001).

Analysis of PTB associated with HIV by age groups revealed that the highest incidence rates were observed for the 30 to 44 years old group. Meanwhile, the highest incidence rates of PTB without HIV occurred among the 60 and more years old individuals. (Table 1).

**Table 1:** Incidence rates of Pulmonary TB, according to age groups and HIV diagnosis. Mexican Institute of Social Security, 2006–2014 per 100,000.

	Pulmonary TB with out HIV				Pulmonary TB with HIV			
year/age group	15–29	30–44	45–59	60+	15–29	30–44	45–59	60+
2006	8.3	7.1	13.4	15.8	0.39	0.48	0.45	0.16
2007	8.7	7.9	14.3	16.6	0.75	0.57	0.78	0.39
2008	8.8	7.5	14.1	15.4	0.66	0.93	0.77	0.63
2009	8.1	7.0	12.4	13.8	0.37	0.53	0.62	0.35
2010	9.6	7.3	15.1	16.2	0.58	0.54	0.75	0.82
2011	9.8	7.8	14.2	15.8	0.51	0.70	0.53	0.15
2012	9.2	8.0	13.9	15.7	0.25	0.51	0.26	0.19
2013	9.1	7.4	14.2	14.4	0.31	0.68	0.36	0.16
2014	9.3	7.5	13.1	14.9	0.30	0.60	0.24	0.08

The characteristics of tuberculosis patients are shown in Table 2, we observed that although there are differences in the proportion of PTB cases by region was not for HIV + PTB patients. Of the 196 cases of pulmonary tuberculosis with HIV / AIDS that died, 17 of them the main cause of death was pulmonary tuberculosis, the remainder was for another reason that we specifically ignore, due to the design of the registry of death in the special system of epidemiological surveillance. A statistically significant difference for the completion of treatment and healing between the two groups, also in the proportion of death during treatment was found.

**Table 2:** Characteristics of pulmonary TB patients according to HIV diagnosis. Mexican Institute of Social Security, 2006–2014.

Characteristic	Total	Pulmonary TB with HIV	Pulmonary TB without HIV	p-value*
	n = 31,352	n = 1,325	n = 30,027	<0.001
	Number / (%)	Number/total (%)	Number/total (%)	
**Female**	12,937 (41.3)	356 (26.9)	12,581 (41.9)	<0.001
**Male**	18,415 (58.7)	969 (73.1)	17, 446(58.1)	<0.001
**Age (years) [median(IQR)]**	49 (32–62)	39 (30–51)	49 (32–63)	0.02**
**Grup age**				
**15–29**	6,687	318 (4.8)	6,369 (95.2)	p<0.001
**30–44**	6,794	514 (7.6)	6,280 (92.4)	p<0.001
**45–59**	8,481	305 (3.6)	8,176 (96.4)	p<0.001
**60+**	9,390	188 (2.0)	9,202 (98.0)	p<0.001
	p<0.001	p<0.001	p<0.001	
**Region**				
**Northern region**	15,694 (50.1)	466 (35.2)	15,228 (50.7)	<0.001†
**Central region**	7,017 (22.4)	436 (32.9)	6,581 (21.9)	<0.001†
**Southern region**	8,641 (27.6)	423 (31.9)	8,218 (27.4)	<0.001†
	p<0.001	p = 0.322	p<0.001	
**Treatment outcome**				
**Cure and treatment completion**	26,897 (85.8)	1005 (75.9)	25892 (86.3)	<0.001†
**Failure**	278 (0.9)	13 (1.0)	265 (0.9)	0.375†
**Default**	1,459 (4.7)	74 (5.6)	1,385 (4.6)	0.100†
**Death during treatment**	1,973 (6.3)	196 (14.8)	1,777 (5.9)	<0.001†
**Other**^a^	745 (2.3)	37 (2.7)	708 (2.3)	0.309†

*Chi-square test.

**Mann–Whitney Test.

^†^Binomial test.

^a^ The category of "other" includes registered cases as they continue in treatment, transfers and the status was ignored.

We perform a binary logistic regression using as dependent variable PTB with HIV patients and as covariates age, sex and region, finding association in each of them, the results are shown in Table 3.

**Table 3:** Association of pulmonary TB with HIV and patient characteristics by multivariate analyses. Mexican Institute of Social Security, 2006–2014.

Variable	Adjusted OR	(95% CI)	p-value
Age (years)	0.976	(0.973-.979)	<0.001
Male	2.059	(1.818–2.332)	<0.001
Region			
Northern region	0.55	(0.48–0.63)	<0.001
Central region	1.464	(1.273–1.683)	<0.001
Southern region	1		

We analyzed the association between PTB with HIV and treatment failure. Characteristics of patients who failed treatment as compared to those of patients who were cured or completed treatment and adjusted analyses for age, sex and region the associated to treatment failure are shown in table 4. We did not find statistically significant differences between treatment failure and PTB patients with HIV and without HIV. The treatment failure was associated with sex of the patient and the central region.

**Table 4:** Association of treatment failure with HIV and other patient characteristics with pulmonary TB results, by multivariate analyses 2006–2014.

Variable	Adjusted OR	(95% CI)	p-value
HIV	0.743	(.422–1.308)	0.303
Male	1.316	(1.027–1.686)	0.03
Age (years)	1.008	(1.002–1.015)	0.15
Region			
Northern region	0.783	(.604–1.014)	0.783
Central region	0.432	(.297-.629)	<0.001
Southern region	1		

We also looked at the association between PTB patients without HIV and death from any cause during the treatment, for it conducted a multivariate analysis through a binary logistic regression including the following variables: age, gender, and region. The results in Table 5, where a strong association between HIV and the probability of dying was observed.

**Table 5:** Association of death with HIV and other patient characteristics with pulmonary TB results, by multivariate analyses 2006–2014.

Variable	Adjusted OR	(95% CI)	p-value
HIV	4.001	(3.381–4.734)	<0.001
Male	1.458	(1.320–1.611)	<0.001
Age (years)	1.041	(1.038–1.044)	<0.001
Region			
Northern region	1.301	(1.162–1.456)	<0.001
Central region	0.956	(.835–1.093)	0.509
Southern region	1		

## Discussion

TB and HIV present particular challenges to adherence. Both are chronic and infectious diseases that affect mainly the most disadvantaged populations and involve complex treatment regimens with potentially severe side effects; both are public health priorities and non-adherence may cause drug resistance. Treatment adherence is also affected by beliefs about the origins, transmission and treatment of TB and HIV, often resulting in the stigmatization of those affected [9].

As in other studies revealing decreased PTB in Mexico [10], we found that the incidence rate of PTB has decreased in the IMSS. The possibility of under PTB notification cases are very low because the treatment is only given if the case is notificate, the treatment is free for all cases.

People without HIV / AIDS have a tendency to increase in young people, while in people without the virus it is decreasing. The following may be explained by the fact that there are more people with active and latent tuberculosis in the general population than the population living with HIV / AIDS (Table 1).

The overall incidence rate of PTB with HIV in the period decreased, this may be associated with decreased HIV cases in the Latin America Region [11]. In Latin America, the number of new HIV infections in 2014 was 17% lower than in 2000, however in this study the incidence rate increased slightly in women. These findings reflect a number of complex phenomena that reveal the social and biological vulnerability of women; resulting in a feminization of the epidemic; but also with an increased awareness of women and health personnel to detect this disease. Also from the psychological point of view, women bear greater guilt, which affects their self-esteem and suffer periods of depression. So the approach of care is complex, according to consider the realities and contradictions of every woman. The feminization of the HIV epidemic in Mexico is not new, according to data from the Secretary of Health revealed in the 1990s, he had infected one woman for every six men, and while for 2007, it was one woman for every four men [12].HIV transmission among women is primarily sexual, with the apparent practice of heterosexual partner without sexual protection [13]; sexuality so happens has to be perceived not for reproductive purposes, but of desire.

The incidence rate of PTB with HIV in men is 3.35 times higher than women. Furthermore, our data suggested that patients suffering from both conditions (PTB and HIV) have no difference in the probability of failure of treatment contrary to other reports [14–15].Hypotheses to this is adherence to tuberculosis treatment with people living with HIV/AIDS, detection of PTB in patients suffering from HIV/AIDS or PTB patients on antiretroviral therapy were more likely to have successful treatment outcomes than those not on antiretroviral therapy (ART). Also, it has been documented that despite availability of free ART from health institutions, mortality was high among TB-HIV co-infected patients, and strongly associated with the absence of ART during TB treatment [16].Also, because it is conceived that generally people living with HIV and benefit from receiving antiviral treatment, does not guarantee a relapse; since may remain in a "healthy" state if you are disciplined with much labor time awareness.

Also, it has been documented the effectiveness of the current approach to the treatment of tuberculosis patients regardless of HIV status [17],what is reflected in this study. This phenomenon can be interpreted from various theoretical approaches, such as: 1. Behavioral (learning) perspective, is focused on the environment and the teaching of skills to manage adherence [9]. Protection motivation theory, based on the motivation to engage in preventive behavior [18]. Social-cognitive theory, is focused the knowledge of health risks and benefits are a prerequisite to change, additional self-influences are necessary for change to occur [19] and a self-regulation perspective, based in it is necessary to examine *“individuals”* subjective experience of health threats to understand the way in which they adapt to these threats [20]. However, a major constraint was that we did not capture data of health behavior theories.

As reported in other studies where mortality from PTB is associated male sex [21], we found this association to interact with HIV.

In the present study, we found that PTB and HIV increases the probability of dying during treatment compared to the cases of PTB without HIV, consistent with published other study HIV increases the mortality rates associated with PTB [22]. The percentage of deaths from PTB in HIV treatment is similar to that described by Garcia, et al [23] with 5.8%, whereas in our study was 6.3%. However, it has also been reported that people living with HIV/AIDS are co-infected with tuberculosis, after 50 weeks of follow up, the most frequent causes of death were non-HIV related or tuberculosis related, including drug toxicity [24], this situation could not be measured in the present study. Beyond these results, it is important achieving adherence to TB medication may be seen as an urgent issue for public health because of its infectiousness, and the recent emergence of extremely drug resistant strains [25].

There are a variety of factors in the various realities of people affected with PTB and HIV/AIDS; in a quantitative study may cover the contradictions, circumstances and details among regions; also would consider a very bold the generalization of scientific practice in everyday life of people living with HIV/AIDS and tuberculosis. In this sense, Bauman conceives tuberculosis, a disease *“which ate the body from within as was the poorest “producers” of solid modernity used to die of*.*”* [26].

It is important that the treatment failure was associated with sex of the patient and the central region, whenever the center (especially in Mexico City) is where the greatest health services infrastructure; but it is also where note inequality gaps, which reinforces the conditions of society they are perceived as "something" sick society, which undermines justice; which they are really social distortions, which have in common with the disease, which distort or restrict life chances [27].

For the PTB and HIV, of the key role of preventive therapy as part of a comprehensive control strategy for tuberculosis must be recognized and executed [28]. Many studies propose isoniazid; however in our country only 2.4% of cases had prophylaxis [29]. So it is a wakeup call to study and prescribe chemoprophylaxis in people who deserve it. So the binomial care prescription recommended is increasing access to ART and isoniazid preventive therapy and co-location of HIV and TB treatment [10].

A major limitation of the study were that the determination of living with HIV/AIDS was as self-report; clinical stage of HIV/AIDS while diagnosing the PTB, specific treatment against HIV/AIDS and CD4 cell count level were ignored.

### Conclusions

There was not association between PTB with HIV and treatment failure, and PTB and HIV increases the probability of dying during treatment compared to the cases of pulmonary tuberculosis without HIV; given the limitations of the study, it is necessary to conduct prospective studies that consider specific variables of HIV / AIDS, in order to provide greater precision.
